# Impact of Acorn Flour on Gluten-Free Dough Rheology Properties

**DOI:** 10.3390/foods9050560

**Published:** 2020-05-02

**Authors:** R. Beltrão Martins, M. C. Nunes, L. M. M. Ferreira, J. A. Peres, A. I. R. N. A. Barros, A. Raymundo

**Affiliations:** 1 CITAB—Centre for the Research and Technology of Agro-Environmental and Biological Sciences, Universidade de Trás-os-Montes e Alto Douro, 5000-801 Vila Real, Portugal; lmf@utad.pt (L.M.M.F.); abarros@utad.pt (A.I.R.N.A.B.); 2 CQVR—Chemistry Research Centre, Chemistry Department, Universidade de Trás-os-Montes e Alto Douro, 5000-801 Vila Real, Portugal; jperes@utad.pt; 3 LEAF—Linking Landscape, Environment, Agriculture and Food, Instituto Superior de Agronomia, Universidade de Lisboa, Tapada da Ajuda, 1349-017 Lisbon, Portugal; crnunes@gmail.com (M.C.N.); anabraymundo@isa.ulisboa.pt (A.R.)

**Keywords:** acorn flour, gluten-free dough, fibre-rich ingredient, underexploited resources, rheology, pasting properties

## Abstract

Gluten is a fundamental ingredient in breadmaking, since is responsible for the viscoelastic behaviour of the dough. The lack of gluten has a critical effect on gluten-free dough, leading to less cohesive and less elastic doughs, and its replacement represents a challenge for bakery industry. However, dough rheology can be improved combining different ingredients with structural capacity and taking advantage from their interactions. Although acorn flour was used to bake bread even before Romans, nowadays is an underexploited resource. It presents good nutritional characteristics, particularly high fibre content and is naturally gluten free. The aim of this study was to use acorn flour as a gluten-free ingredient to improve dough rheology, following also market trends of sustainability and fibre-rich ingredients. Doughs were prepared with buckwheat and rice flours, potato starch and hydroxypropylmethylcellulose. Two levels of acorn flour (23% and 35% *w*/*w*) were tested and compared with control formulation. Micro-doughLAB was used to study mixing and pasting properties. Doughs were characterised using small amplitude oscillatory measurements (SAOS), with a controlled stress rheometer, and regarding Texture Profile Analysis (TPA) by a texturometer. Dietary fibre content and its soluble and insoluble fractions were also evaluated on the developed breads. Acorn flour showed promising technological properties as food ingredient for gluten-free baking (improved firmness, cohesiveness and viscoelasticity of the fermented dough), being an important fibre source.

## 1. Introduction

Gluten is one of the most important ingredients in bread making. It is responsible for the viscoelastic behaviour of the dough [1]. Gluten matrix has a major role on the main dough properties: extensibility, stretching resistance, mixing tolerance, gas-holding capacity and allows to obtain a high-quality bread crumb structure [2].

On the other hand, gluten-free dough (GFD) is unable to form a protein network similar to gluten. This raises several difficulties and thus the loss of quality on gluten-free bread baking [3]. The lack of gluten has a critical effect on dough rheology (less cohesive and elastic than wheat dough), formulation process and sensory quality of the final product [2]. The replacement of gluten in baking is a great challenge. There is no raw material or any other ingredient capable of completely replacing gluten, in terms of structural builder. Only the combination of different ingredients and its interactions, with adequate technologies, can improve gluten-free bread (GFB) quality [3,4,5,6]. Therefore, innovative ingredients with structural properties, novel approaches and processes, have been studied and tested with notable results so far obtained [7,8,9,10,11]. In our study, a mixture of natural gluten-free flours was used: buckwheat flour, rice flour, potato starch, acorn flour, and hydroxipropylmethylcellulose (HPMC) as thickening agent.

Rheology measurements are essential tools to understand the performance of the doughs, allowing to predict the final result and the before quality of bread [12]. Among the different rheological techniques, the small amplitude oscillatory shear (SAOS), demonstrated to be adequate to characterise viscoelastic materials and its structure properties [13]. The rheology of GFD, is also very important in order to understand the doughs behaviour during the baking process. Although the studies about this subject have recently increased, the information available is still limited [14]. Additionally, the majority of the GF flours are starch based, yet with different botanical sources, leading to completely diverse performances, according to the GF dough formulation [15].

Nowadays, due to resource scarcity worldwide, is essential to use by-products and underexploited natural raw materials that are not valued enough in food production. This fact led to market trends of sustainability [16], natural products and fibre-rich ingredients, which have contributed to the introduction of underexploited natural raw materials into food chain [17]. Acorn flour is an example, in particular to special requirements products as GF bread [18], increasing the importance of researching this flour. Several authors have been recently studying the incorporation of acorn flour in gluten containing biscuits and bread, in combination with wheat and barley. In all studies, the incorporation of acorn flour until a specific proportion improved the quality of the different final products, both in nutritional and sensorial characteristics [19,20,21]. Korus et al. [22] and Skendy et al. [11] have studied the effect of acorn flour addition in GF bread making, and both concluded that acorn flour incorporation in GF bread could be useful for nutritional and technological reasons. Additionally, many other innovative GF flours, and their impact on GF bread making, have been studied. Particularly, alternative sources of protein, like insects and legumes, found to improve GF bread nutritional composition and contribute to substitute gluten role [23,24]. In addition, microalgae, sorghum, millet, pseudo-cereals, fruit-based flours, by-products and other naturally GF flours have been tested, with very promising results [5,8,9].

Acorn is the fruit of *Quercus* genus tree. These trees are the basis of the Sustainable Agriculture System named “*Montado*” in South of Europe. In Portugal, acorns are abundant and can be considered an underexploited resource, since nearly 50% of the fruits in the trees are not used or harvested [17]. Many authors [25,26] state the use of acorn flour to bake bread has occurred since before the Roman Era. Veiga de Oliveira et al. [27] go further, referring that this flour was already consumed even before the Romans [27]. This fruit was traditionally present in the Mediterranean diet, some decades ago, especially in scarcity years [26]. The acorns most commonly used in human feed were from *Quercus ilex* and *Quercus rotundifolia* (holm oak). Since they are not so bitter comparing to other species, is not necessary to remove bitterness, as Korus et al. [22] described in their work. According to Silva and co-workers [18], acorn flour has interesting nutritional characteristics, and is rich in lipids (8.5%), especially in unsaturated fatty acids and fibre (9.5%–11.5%). Fibres have a major role in GFD rheology characteristics, besides improving nutritional GF bread quality [28,29].

Comparing this work with the referred authors [11,22] who have studied acorn flour incorporation in GF bread, it is important to highlight the innovation of our study. Since holm oak acorn is naturally slightly sweet, no bitterness removal treatment is needed, as mentioned before, which is a great technological advantage. The amount of water added to each flour blend was assessed through Micro-DoughLAB tests. Bread control formulation has buckwheat flour besides starch-based flours. Finally, pasting properties and dough TPA measurements were performed.

The objective of this work was to study the impact of acorn flour from holm oak, on the rheology characteristics of the GF dough, and also on the dietary fibre content, both soluble and insoluble fractions, of the GF baked breads. Two levels of incorporation (23% and 35%) were considered.

## 2. Materials and Methods

### 2.1. Raw Materials

Doughs formulations were prepared with the following flours: buckwheat flour (Próvida, Pêro Pinheiro, Portugal), acorn flour from dried holm oak (*Quercus ilex* and *Quercus rotundifolia*) (Terrius, Marvão, Portugal), rice flour (Fábricas Lusitana, Castelo Branco, Portugal), potato starch (Colmeia do Minho, Paio Pires, Portugal), dried yeast (Fermipan^®^, Lesaffre, Marcq-en-Baroeul, France), hydroxipropyl methylcellulose (HPMC) as a gelling agent (Wellence^TM^ 321, Dow, MI, USA), sugar, sunflower oil, salt, and water. The flour ingredients and the HPMC were kindly supplied for free, except yeast, sugar, oil and salt that were purchased from local market.

### 2.2. Dough Preparation and Sampling

Fernandes [30] developed and analysed a GFD recipe that showed promising results, based on buckwheat flour, rice flour and potato starch, with HPMC as a gelling agent. The control dough (C) was prepared without acorn flour addition. Preliminary assays were conducted to adjust the water content of the control formulation to produce breads having the best quality, based on bread volume and crumb firmness, by testing different water hydrations (data not presented). Two incorporation levels of acorn flour were tested, 23% *w*/*w* (sample A23%) and 35% *w*/*w* (sample A35%) of total flours mixture, respectively, 50% and 75% in relation to the buckwheat flour. The three formulations studied are summarised in Table 1.

Micro-doughLAB 2800 (Perten Instruments, Sidney, Australia) manufacturer’s protocol at 63 rpm speed (AACC (American Association of Cereal Chemists) Method 54-21.01) was used to assess the optimum water absorption capacity for formulations with acorn flour. The peak value of torque of the optimised control-formulation was used as a reference (93 mN.m). Optimum water absorption values (amount of water needed to achieve target peak torque, corrected to 14% moisture basis) were determined for the formulations containing acorn flour in order to achieve a peak of 93 mN.m ± 4%.

The moisture of the different flours was measured through an automatic moisture analyser PMB 202 (Adam Equipment, Oxford, CT, USA). The values were buckwheat flour—12.35%; acorn flour—8.03%; rice flour—11.08%; potato starch—18.10%.

Ingredients were mixed in a Thermo processor equipment (Bimby—Vorwerk, Wuppertal, Germany), initially to activate the yeast, by adding water, yeast and sugar for 2 min at 27 °C, at velocity 1. Following, the other ingredients were added and mixed for 10 min in a dough mixing program (wheat ear symbol). The resulting dough was divided in three portions and poured into three equal containers and placed in a fermentation chamber Arianna XLT133 (Unox, Cadoneghe, Italy) for 50 min at 37 °C. All measurements were carried out after fermentation: three loaves of each formulation were prepared, and all the analyses were performed minimum in triplicate: three measurements were taken from independent three portions of each loaf. Mean values and standard deviations from each determination were recorded.

In a parallel work, the impact of acorn flour addition in the bread characteristics was studied, using exactly the same dough formulations above described. After fermentation, the dough was baked in an electric oven Johnson A60 (Johnson & Johnson, NJ, USA) for 50 min at 180 °C. Correlations between dough properties and breadmaking characteristics (bread firmness) were analysed.

### 2.3. Rheology Measurements

#### 2.3.1. Pasting Properties

The pasting properties of flour blends, according to each GFD formulation, were tested using a Micro-doughLAB 2800 (Perten Instruments, Sidney, Australia), following the manufacturer’s cooking protocol with some modifications [31]. Constant mixing rate at 63 rpm for 43 min (2580 s), and the following temperature cycle: 30 °C for 6 min, then rise temperature until 90 °C during 15 min, the temperature of 90 °C keeps constant for 7 min, then decrease to 50 °C during 10 min and finally, constant temperature of 50 °C for more 5 min, until the end. Only the flours and HPMC were added in the mixture, and 4.00 ± 0.01 g of each flour blend sample was weighed and placed in the chamber of Micro-doughLAB. The amount of water used was studied in previous mixing tests to find the optimum water absorption. Additionally, pure buckwheat flour and acorn flour were tested in the same conditions. Tests were performed at least in triplicate. Concerning rice flour and potato starch, since these ingredients were in the same share in all the formulations, were not submitted to pasting test.

#### 2.3.2. Small Amplitude Oscillatory Measurements (SAOS) Testing

For dough characterisation, by dynamic rheometric measurements, formulations were prepared with all ingredients, as previously described. Rheological tests were performed using a controlled stress rheometer (MARS III, Haake, Germany) coupled with a UTC-Peltier system. A serrated parallel-plate P20 (diameter of 20 mm) was used, with a gap between plates of 1 mm. Each sample was prepared in a small portion of dough, and after 50 min of fermentation at 37 °C in a fermentation chamber Arianna XLT133 (Unox, Cadoneghe, Italy), was allowed to rest for 30 min at 5 °C. The fermented dough samples were placed between the plates, and after its adjustment, the edges were covered with paraffin to protect the sample from dehydration during measurements. Rheometer temperature was kept at 5 °C during test performance to avoid dough fermentation. Samples were left for 15 min before the test, to allow residual stress to relax and to stabilise the temperature. All rheological measurements were run, at least, in triplicate.

Small amplitude oscillatory shear (SAOS) tests were conducted to assess linear viscoelastic properties of the GFD. The dynamic linear viscoelastic region of each sample was previously determined through stress sweep tests at frequency of 6.28 rad/s (1 Hz). Frequency sweeps (from 0.0628 to 628 rad/s) were performed at constant shear stress of 10 Pa.

#### 2.3.3. Dough Texture Profile Analysis (TPA)

Texture profile analysis (TPA) was performed after dough fermentation at 37 °C during 50 min, using a texturometer TA.XT.plus (Stable Micro Systems, Surrey, England) equipped with a load cell of 5 kg and penetration mode of the equipment set up. Dough samples were placed in cylindric containers with 100 mm in diameter and a height of 80 mm. These cylinders were completely filled by the sample.

The measurement conditions were 19 mm diameter acrylic cylindrical probe, 20 mm of penetration distance, 1 mm/s of crosshead speed and 5 s of waiting time between the two measurements cycles. Each measurement was repeated at least four times. Firmness and cohesiveness were the main representative texture parameters obtained from TPA, to characterise the dough, as it was reported by Nunes et al. [8].

### 2.4. Colour Characterisation and pH Values of GF Dough

Dough colour was determined using a colorimeter Croma-Meter CR 400 (Konica-Minolta Sensing Americas, New Jersey, USA), through the CIE L*a*b* system (International Commission on Illumination) using the following parameters: L*—lightness variable (L* = 100 white, L* = 0 black); a*—intensity of green (−60 < a* < 0) or red (0 < a* < +60); and b*—intensity of blue (−60 < b* < 0) or yellow (0 < b* < +60). Each sample was measured in triplicate.

pH measurements were performed in three different points of each fermentation dough, with a potentiometer pH-Meter Basic 20 (Crison Instruments, Barcelona, Spain), until stabilisation.

### 2.5. Dietary Fibre: Soluble, Insoluble and Total Fibre in GF Bread

According to Martins et al. [32], gluten-free breads were prepared from the same GF doughs studied in this work, as previously described. The soluble, insoluble and total fibre of GF breads (Control, A23% and A35%), were determined by using a “Total Dietary Fibre Assay Kit” from Megazyme (Wicklow, Ireland). According to manufacturer’s information, the analysis can be considered to be divided in two methods: (1) based on AOAC (Association of Official Agricultural Chemists) Method 991.43 “Total, Soluble, and Insoluble Dietary Fibre in Foods” (First Action 1991) and AACC Method 32-07.01 “Determination of Soluble, Insoluble, and Total Dietary Fibre in Foods and Food Products” (Final Approval 10-16-91); (2) determination of total dietary fibre based on AACC method 32-05.01 and AOAC Method 985.29. All procedures were done at least in duplicate.

### 2.6. Statistical Analysis

Results were analysed using the statistical programme Origin Pro 8 (OriginLab Corporation, MA, USA). Experimental data were compared using analysis of variance (one-way ANOVA), and the Tukey test was used to evaluate mean differences at a confidence level of 95%, with significant differences considered to be *p* < 0.05. Results are presented as mean values and standard deviations. Correlations were tested between the different variables, using the program Statistica 10.0 and the function Bivariate Scatterplot.

## 3. Results and Discussion

### 3.1. Dough Rheology Characterisation

#### 3.1.1. Pasting Properties

**Figure 1:**
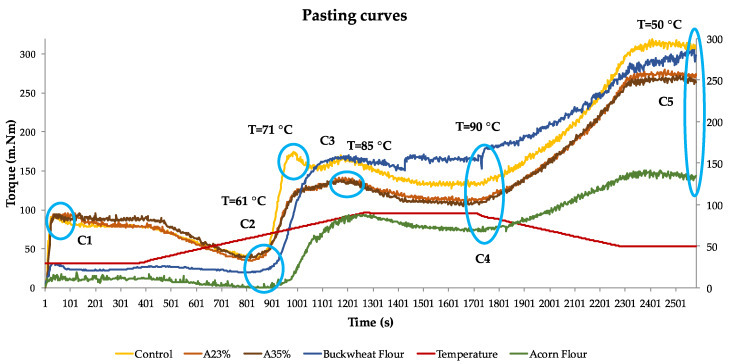
Pasting curves of the tested blend flours: control, A23% and A35%, and also buckwheat and acorn flour.

Flour blends’ and pure flours’ pasting curves, obtained from the MidrodoughLab are represented in Figure 1, with the main points marked with a blue circle.

In Figure 1, it is possible to observe the maximum torque of the flour blends’ mixing curves, that is represented in C1, with a constant temperature of 30 °C. After 6 min, the temperature starts rising. The graphic shows the breakdown (C2), minimum torque (linked with minimum viscosity) at 61 °C, corresponding to protein weakening. All the flour blends had a similar response concerning both temperature and torque (40 m.Nm). At the same stage (C2), pure flours showed the minimum torque, related with a minimum of viscosity, at the same temperature, but with a lower torque comparing with the blends. Buckwheat flour torque (directed related with its viscosity) was around 20 m.Nm and acorn flour dropped to 1 m.Nm, which is in agreement with [33], that found likewise, lowest viscosity in the breakdown for acorn starch comparing with buckwheat.

Along the heating process, the starch granules retain water and swell, which results in viscosity increasing, until peak viscosity (C3) is reached and indicates water-binding capacity, and starch gelatinisation [34,35]. Control flours mixture reached the gelatinisation peak torque (175 m.Nm) at 71 °C and both acorn flour mixtures at 85 °C, but with a lower torque (137 m.Nm). From the curve of acorn flour, with a lower peak torque (87 m.Nm), it is possible to understand this behaviour, where the contribution of acorn flour decreased the viscosity of the blends A23% and A35%. Buckwheat flour presents higher viscosity, as well as the control blend, where this flour is present in 46% share. In GFD, starch gelatinisation performs an essential function, since it has the aptitude to trap gas bubbles in its own matrix, promoting the gas holding capacity of the dough [36,37].

Starch gelatinisation depends on the starch characteristics and its swelling capacity. Moreover, the starches establish interactions with the HPMC, a fact that also contributes to the maximum viscosity of the dough, reached during gelatinisation [15]. According to Hager et al. [38], buckwheat flour has approximately 60% of starch, with around 25% amylose, while acorn flour has around 50% of starch [18], where 55% is amylose [39]. Singh et al. [40] state that amylose and lipid contents are one of the causes for the differences between the swelling capacity of starches. Higher lipid content is supposed to decrease the swelling capacity of individual granules [40]. However, Debet and Gindley [41] suggest that when the amylose content is high, the effect of lipids is secondary, concluding that high amylose content inhibits swelling. Correia et al. [39], concluded fewer amylose leaching and lower degree of gelatinisation has been attributed to acorn structure type of starch. Regarding the higher temperature of acorn flour gelatinisation, according to Correia et al. [39], indicates increased resistance to swelling, a characteristic response of acorn starch. On the contrary, according to Debet and Gindley [41], higher viscosity reveals starch with higher swelling capacity, and Torbica et al. [42] identify it as a pasting property of buckwheat flour.

The following stage is characterised by a viscosity decreasing to a minimum (breakdown). This happens when the granule absorbs as much water as to achieve its rupture point, while temperature keeps increasing [40]. As we can see from the results, the breakdown (C4) occurs at the end of 90 °C with higher torque (132 m.Nm) for control dough and lower torque (110 m.Nm), meaning lower viscosity, for both acorn blends. Buckwheat flour showed the highest torque (154 m.Nm) and acorn the lowest (68 m.Nm), revealing the contribution that each pure flour has in the respective blends, according to the different levels of incorporation in the dough.

When gelatinised starch temperature cools, viscosity increases until the end of the test (C5), the moment when we can see the formation of a gel, resulting from amylose retrogradation [34]. Regarding Figure 1, is possible to observe that retrogradation torque was quite similar between control blend (305 m.Nm) and pure buckwheat flour (300 m.Nm). Following, the acorn flour blends, present a lower torque, respectively, A23% (270 m.Nm) and A35% (262 m.Nm). Finally, pure acorn flour torque was the lowest (129 m.Nm). Despite this retrogradation low viscosity of acorn flour, it is possible to recognise that acorn flour blends have a quite high viscosity, when comparing with the pure flour. Synergetic effects between the starches, proteins, HPMC and fibres, and also the proportion of acorn flour in the blends, can explain why these blends are able to maintain a higher viscosity in the retrogradation process. Paste retrogradation is influenced by the amylose content and also by the structural arrangement of the starch chains [37,38]. According to Hager et al. [35], buckwheat starch presents a granular shape, whereas acorn starch granules show a spheroid/ovoid and cylindrical shape [43].

Since our study is focused on acorn flour, it is important to highlight that among the different species of oak trees, the acorn starch is also different due to taxonomic characteristics of the plant [43]. For this reason, to make possible the comparison about starch behaviour, we used the studies based on the same species: *Quercus rotundifolia* and *Quercus ilex* from Cappai et al. [43] and Correia et al. [39].

Finally, it is also important to mention the role that proteins have in the dough rheology behaviour, contributing as well to gluten replacing functions [3]. Different authors [41,44,45] state that interactions between the different types of starch and proteins, have an influence on the dough rheology characteristics, affecting also pasting properties of starch. Concerning the developed doughs, the average protein content (data obtained from suppliers) of buckwheat is 12%, and acorn flour is 4.5%, in accordance with literature [18,38]. As previously described in Section 2.2, samples A23% and A35% have 50% and 75% replacement of buckwheat flour, by acorn flour, respectively, when comparing with the control sample. This means that buckwheat flour changes from 46% incorporation in control to 23% in A23% sample and to 11% in A35%, with a corresponding increasing of 23% and 35% of acorn flour. In relation to protein, acorn flour presents approximately 8% less content than buckwheat flour, resulting in a lower protein content on sample blends A23% and A35%. This will influence protein–starch interactions and consequently pasting properties. As we can observe from the graphic, torque values in C3, C4, and C5, are lower in acorn flour blends when comparing to the control blend. Similar results have been reported in different studies about proteins influence in starch pasting properties [46,47,48].

#### 3.1.2. Small Amplitude Oscillatory Measurements (SAOS)

**Figure 2:**
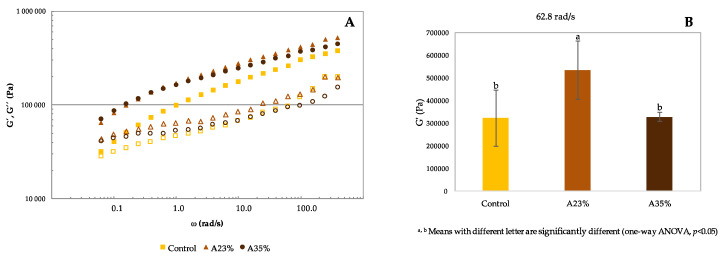
(**A**): Mechanical spectra of GF doughs (control, A23%, A35%); G´ (storage modulus—filled symbol), G′′ (loss modulus—open symbol). (**B**): G′ values at 62.8 rad/s (10 Hz) for the same samples.

The mechanical spectra of the GF doughs (control, A23%, A35%) are presented in Figure 2A. The mechanical spectra of GF doughs represent the variation of storage modulus (G′) and loss modulus (G′′) as a function of angular frequency (ω). In order to promote a more systematic comparison of the spectra, the values of G′ obtained at 62.8 rad/s (10 Hz) are presented in Figure 2B.

It is possible to observe in Figure 2A, that both moduli G′ and G′′ show high frequency dependence. The three GF doughs compared presented higher values of G′ in comparison with G′′, in the whole frequency ranges, expressing a predominance of the elastic behaviour. It is also important to note that the control dough shows lower values of the viscoelastic functions and a pseudo-terminal region at low frequency values, with a tendency for a crossing point at very low frequencies. This type of behaviour reflects the existence of a less structured system in the control dough, which may be associated with a less stable structure [22]. In other words, the incorporation of acorn flour had an important role in increasing the degree of structuring of the dough. This increase in the degree of structure should have resulted from the greater ability of acorn starch to create structures, which was evidenced by the results obtained in terms of pasting curves. Our results are in accordance with different authors [22,49,50] that also have studied GF dough with different botanical source flours (respectively, acorn flour, carob flour and legume flours), and obtained similar results, where G′ > G′′, showing a soft gel-like structure.

About G′ values at 62.8 rad/s, a significantly higher value (*p* < 0.05) for A23% dough was observed, meaning that the addition of acorn flour in dough formulation induced a significantly increase of elastic modulus, acting as a strengthening agent of the structure. However, the dough with 35% of acorn flour, reduces significantly G′ values, being similar to control dough. A similar effect will be found in the texture results, in terms of the TPA parameters. This fact indicates that the growing incorporation level of acorn flour presents a negative impact on the structure, since it is possible to observe a modification of the rheological behaviour, with the reduction of the elastic modulus at 62.8 rad/s. The aforementioned behaviour is not significantly different (*p* > 0.05) from the one of control dough, showing that the benefits, in terms of viscoelastic characteristics, induced from the addition of acorn flour are not extend to higher levels of incorporation. Regarding the improvement of rheology characteristics observed in acorn flour doughs, it is also important to underline the contribution of the fibre content present in acorn flour. Several authors have studied the impact of fibre in GFD formulation, with the objective of improving rheology. The dough formulation with the inclusion of fibre-rich ingredients, positively influenced rheological properties, with higher values of both moduli, and structuring the GF dough closer to a gel-like material [11,28,35,51,52].

Korus et al. [22] obtained different results, where partial (20%, 40%, 60%) replacement of starch with acorn flour, resulted in a significant increase in both moduli, and proportional to the extent of acorn flour replacement. Nevertheless, it is important to clarify that the control dough formulation was based on corn and potato starch, totally different from the control dough in our study. Nevertheless, the author refers that higher levels of acorn flour incorporation revealed a negative impact, since it caused the reduction of loaf volume, not shown in rheology tests [22]. This should also be related with a negative impact on the dough viscoelasticity.

#### 3.1.3. Texture Profile Analysis (TPA)

**Figure 3:**
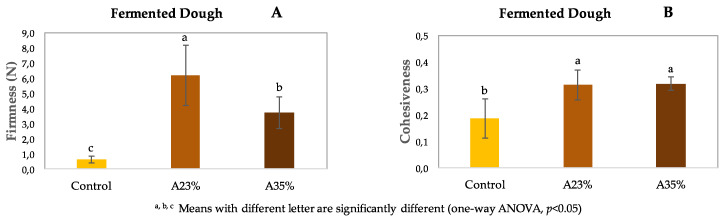
Firmness (**A**) and cohesiveness (**B**) of GF doughs (control, A23% and A35%).

Figure 3 shows the results of TPA: firmness and cohesiveness. The incorporation of both acorn flour levels, 23% and 35%, increased significantly (*p* < 0.05) the firmness of the dough comparing with control. Between the two acorn flour doughs, the firmness is significantly (*p* < 0.05) higher in A23% when comparing with A35%. For the lowest level of incorporation, it was possible to obtain a more structured dough, already supported in terms of increasing the elastic modulus (G′), which corresponds to a higher firmness value. For the 35% of acorn flour incorporation, the extension of the network developed is weaker, resulting in a softer dough, similar to the control. Probably, the excess of acorn starch caused the weakening of this network, as we can observe from pasting curve analysis, where a noticeable decrease of acorn flour viscosity parameters is revealed.

These findings are in accordance with the previous rheology measurements results discussion. Nevertheless, the firmness of the dough with 35% of acorn flour is significantly higher than the control dough. The results show that partial replacement of buckwheat flour by acorn flour improves the firmness of the dough, for either levels of acorn flour.

Cohesiveness is associated with the level of structure between the different elements that take part in the dough matrix [53]. The obtained results demonstrate that dough A23% and also dough A35% significantly increased (*p* < 0.05) their cohesiveness, when compared with control dough. However, no significant differences were found between the two levels of acorn flour incorporation. From these results, it is possible to observe that the incorporation of acorn flour in the formulation, with the reduction of buckwheat flour share, increases the cohesiveness of the dough. Furthermore, Correia et al. [39] also concluded that acorn starches have the ability to contribute to a coherent structure.

The increase of firmness and cohesiveness of the dough with acorn flour addition, together with the reduction of buckwheat flour incorporation level, can be explained due to the nutritional composition of acorn flour [18] in comparison to buckwheat flour [38]. As previously mentioned, acorn flour has a higher content of fibre (9.5%–11.5%) and total fat (8.5%), when compared to buckwheat flour with about 2% of fibre and 5% of total fat [18,38]. According to different authors presented in a review article, the level of fibre has a major role as structure enhancer in GF leavened bread, as well as the interactions between the different components of the complex dough system. Due to water binding capacity, gel forming ability, fat mimetic, textural and thickening effects, fibres have a positive impact on the texture, improving both firmness and cohesiveness [52]. As previously reported by Graça et al. [7], firmness and cohesiveness are the most representative texture parameters of GF dough, as they have a great influence on bread quality and acceptance [3].

### 3.2. Impact of Acorn Flour Addition in Bread Texture

**Figure 4:**
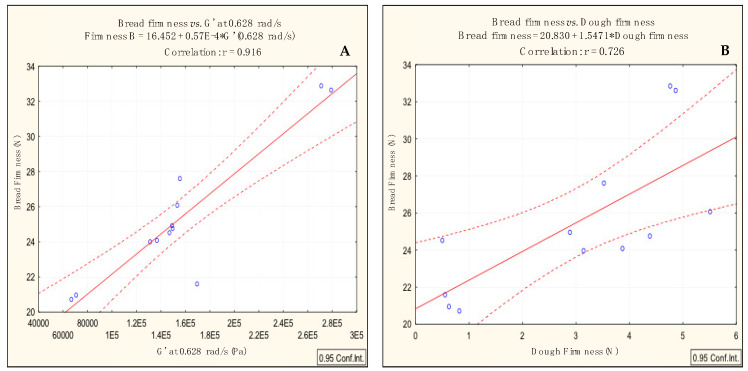
(**A**): Correlation between bread firmness and the viscoelastic characteristics of the dough (G′ at 0.628 rad/s) and (**B**): correlation between the bread firmness and dough firmness.

In a parallel work, the impact of acorn flour addition in the bread texture was characterised, using exactly the same formulations and processing conditions used in the present study. In that work, it was found that acorn flour incorporation, generally, induces an increase in bread firmness. Through the results obtained in the present study, it was possible to establish a positive correlation between the bread firmness and the viscoelastic characteristics of the dough—G′ at 0.628 rad/s (Figure 4A) and between the bread firmness and the dough firmness (Figure 4B).

The obtained correlations, which allow to predict the behaviour of the bread, in terms of firmness, taking into account the dough rheological behaviour and its texture, are extremely useful in terms of the gluten-free bakery industry. Similar correlations have been obtained by other authors [7,37].

### 3.3. Characteristics of GF Dough: Colour and pH

Table 2 presents the results of GF dough colour measurements and respective pH. It can be observed that pH significantly decreased from control dough to acorn dough. Either levels of added acorn flour led to an acidification of the dough, although there are no significant differences between A23% and A35% (*p* > 0.05).

Concerning dough colour, it is possible to observe that L* value has reduced significantly (*p* < 0.05) with the increase of acorn flour content. These results show a reduction in whiteness and an increase in the browning index, meaning the darkness of the dough. This effect on the dough is important, as it will contribute to the improvement of bread colour. This is a relevant parameter when observing consumers’ preferences. For GF bread, this characteristic is even more important, since it frequently presents pale colours when compared to their wheat counterparts, what is referred as a depreciative factor in GF bread [2]. Regarding the a* and b* parameters of the dough, it is possible to observe a significant (*p* < 0.05) increase with acorn flour incorporation, which means, respectively, a more intensive red than green, and a predominance of yellow over blue. Colour differences (ΔE) between control bread and acorn breads can be calculated by the following equation:

ΔE* = (ΔL*^2^+Δa*^2^+Δb*^2^)^1/2^ (1)

The human eye can only detect the difference in the colours if ΔE > 5 [54]. When comparing the control dough with both acorn flour dough, the obtained results for were ΔE*(control–A35%) = 21.25; and ΔE*(control–A23%) = 15.66, revealing that the difference between the dough colours was really high. Finally, the difference between the two levels of acorn flour dough was ΔE*(A23%–A35%) = 5.61, almost in the human eye threshold perception.

### 3.4. Dietary Fibre: Soluble, Insoluble and Total Fibre

As fibre content increases, besides the rheology improvement above mentioned, is also possible to improve GF bread nutritional profile. Several authors have studied the addition of fibre-rich raw materials in GF bread formulation, obtaining very promising results [52]. Fratelli et al. [55] evaluated and optimised the applications of fibre (psyllium) in GF formulation, and obtained a final recipe with an increase of fibre content from 2.5% to 4% and a decrease of glycaemic response, with good acceptability scores. Rocha Parra et al. [35,51] studied apple pomace as an alternative source for fibre enrichment of GF baked products. Tsatsaragkou et al. [49] concluded that carob flour could be a promising solution to develop high quality formulations of GF bread with a fibre-rich ingredient. Hager et al. [38] analysed the chemical profile of nutrient-dense GF flours, and compared them with wheat flours. In addition, Hager et al. [38] have also studied soluble and total fibre, together with recommended daily intake.

According to AACC [56], dietary fibre (DF) is defined as the carbohydrates (from edible part of plants) which are resistant to digestion and adsorption in the human small intestine, with complete or part fermentation in the large intestine. The term DF comprises polysaccharides, oligosaccharides and associated plant compounds. DF is fractionated into insoluble fibre, linked with intestinal regulation, and soluble fibre associated to serum cholesterol reduction levels and with glycaemic response [38,56].

According to Hager et al. [38], buckwheat flour presents approximately 2% of DF, from which about 23% is soluble fibre. Concerning acorn flour, Silva et al. [18] obtained values between 9.5% and 11.5%. Our results were also similar, with 10% of DF, from which about 9% is soluble fibre (data not shown).

The results of GF bread fibre analysis, soluble fraction, insoluble fraction and total fibre are presented in Table 3. From the presented data, it is possible to observe that the incorporation of both levels of acorn flour increased significantly (*p* < 0.05) the total fibre and insoluble fibre, comparing with control bread formulation. This was expected, since acorn is a good source of DF, particularly in the insoluble fraction [22].

However, it is important to note that the soluble fibre content of the breads significantly decreased (*p* < 0.05), comparing with the control formulation, when acorn flour incorporation was considered. This fact can be explained since the soluble fibre fraction of acorn flour in comparison to buckwheat flour is much lower.

According to Melini and Melini [57], a healthy diet needs to be diverse, balanced and it must assure a high intake of DF. Even though DF health benefits are profusely documented, is frequently concluded that recommended daily intake is not followed by the majority of the consumers [35]. In the case of consumers with special requirements as GF diet, it is even more difficult, since GF products are in general described with a lack of fibre [58].

Following USDA recommendations, the DF Reference Intake is 25 g/day for women and 38 g/day for men. If we consider an ingestion of 100 g of fresh bread per day, the 23% acorn flour bread would serve 7.5 g of fibre. This represents about 30% and 20% of daily adequate DF intake for women and men, respectively, meaning that acorn flour bread can be an important vehicle of fibre, with the possibility to use the respective nutritional claim.

Thus, acorn flour seems to be a very promising ingredient, in order to improve DF content in GF bread.

## 4. Conclusions

Based on the obtained results, it can be stated that acorn flour significantly affects the rheology properties of the doughs. Moreover, acorn flour has an impact on the dough’s mixing and pasting curves and improved the firmness, the cohesiveness and the viscoelasticity of the fermented dough. The 23% of acorn flour incorporation presented better results when comparing with 35%. According to SAOS, GF doughs exhibit a weak gel-elastic like behaviour with G’ values higher than G’’ and frequency dependent. Acorn flour incorporation caused the acidification and increased the darkness of the dough, that will have a positive impact in terms of sensory appreciation of the bread. Thus, acorn flour can be a very promising ingredient, in order to improve both rheological GF dough properties and nutritional GF bread quality, in particular DF content, a really important nutrient in special requirement diets.

## Figures and Tables

**Table 1:** Formulation of the gluten-free dough (GFD) samples and respective codes.

Ingredients (%)	Control (C)	Acorn 23% (A23%)	Acorn 35% (A35%)
Buckwheat flour	46.0	23.0	12.0
Rice flour	31.0	31.0	31.0
Potato starch	23.0	23.0	23.0
Acorn flour	0.0	23.0	35.0
Sunflower oil (in relation to flours)	5.5	5.5	5.5
HPMC (in relation to flours)	4.6	4.6	4.6
Dried yeast (in relation to flours)	2.8	2.8	2.8
Sugar (in relation to flours)	2.8	2.8	2.8
Salt (in relation to flours)	1.8	1.8	1.8
Water absorption (14% moisture basis)	65.0	63.0	62.0

**Table 2:** Colour parameters and pH of GF doughs (control, A23% and A35%).

	Control	A23%	A35%
**pH**	5.35 ^a^ ± 0.04	5.09 ^b^ ± 0.05	5.05 ^b^ ± 0.05
Colour L*	82.49 ^a^ ± 0.42	70.90 ^b^ ± 0.08	66.42 ^c^ ± 0.26
Colour a*	0.56 ^c^ ± 0.21	6.11 ^b^ ± 0.35	7.89 ^a^ ± 0.31
Colour b*	15.50 ^c^ ± 0.33	24.45 ^b^ ± 0.83	27.31^a^ ± 0.65

Mean values with different letters in the same row are significantly different (one-way ANOVA, *p* < 0.05).

**Table 3:** Insoluble, soluble and total fibre of GF breads (in dry matter): control, A23% and A35%.

	Control	A23%	A35%
**Insoluble Fibre (%)**	8.99 ^b^ ± 0.63	11.96 ^a^ ± 0.53	12.46 ^a^ ± 0.92
**Soluble Fibre (%)**	1.32 ^a^ ± 0.38	0.31 ^b^ ± 0.24	0.12 ^b^ ± 0.10
**Total Fibre (%)**	10.31 ^b^ ± 0.65	12.27 ^a^ ± 0.75	12.58 ^a^ ± 0.90

Mean values with different letters in the same row are significantly different (one-way ANOVA, *p* < 0.05).
